# Effect of Cholesterol on the Organic Cation Transporter OCTN1 (SLC22A4)

**DOI:** 10.3390/ijms21031091

**Published:** 2020-02-06

**Authors:** Lorena Pochini, Gilda Pappacoda, Michele Galluccio, Francesco Pastore, Mariafrancesca Scalise, Cesare Indiveri

**Affiliations:** Department of Biology, Ecology and Earth Sciences (DiBEST), Unit of Biochemistry and Molecular Biotechnology, University of Calabria, via P. Bucci 4c, 87036 Arcavacata di Rende, Italy; lorena.pochini@unical.it (L.P.); gilda.pappacoda@gmail.com (G.P.); michele.galluccio@unical.it (M.G.); francesco.pastore92@gmail.com (F.P.); mariafrancesca.scalise@unical.it (M.S.)

**Keywords:** acetylcholine, cholesterol, membrane transport, OCTN1, proteoliposomes

## Abstract

The effect of cholesterol was investigated on the OCTN1 transport activity measured as [^14^C]-tetraethylamonium or [^3^H]-acetylcholine uptake in proteoliposomes reconstituted with native transporter extracted from HeLa cells or the human recombinant OCTN1 over-expressed in *E. coli*. Removal of cholesterol from the native transporter by MβCD before reconstitution led to impairment of transport activity. A similar activity impairment was observed after treatment of proteoliposomes harboring the recombinant (cholesterol-free) protein by MβCD, suggesting that the lipid mixture used for reconstitution contained some cholesterol. An enzymatic assay revealed the presence of 10 µg cholesterol/mg total lipids corresponding to 1% cholesterol in the phospholipid mixture used for the proteoliposome preparation. On the other way around, the activity of the recombinant OCTN1 was stimulated by adding the cholesterol analogue, CHS to the proteoliposome preparation. Optimal transport activity was detected in the presence of 83 µg CHS/ mg total lipids for both [^14^C]-tetraethylamonium or [^3^H]-acetylcholine uptake. Kinetic analysis of transport demonstrated that the stimulation of transport activity by CHS consisted in an increase of the Vmax of transport with no changes of the Km. Altogether, the data suggests a direct interaction of cholesterol with the protein. A further support to this interpretation was given by a docking analysis indicating the interaction of cholesterol with some protein sites corresponding to CARC-CRAC motifs. The observed direct interaction of cholesterol with OCTN1 points to a possible direct influence of cholesterol on tumor cells or on acetylcholine transport in neuronal and non-neuronal cells via OCTN1.

## 1. Introduction

OCTN1 belongs to a small sub-family of organic cation transporters that includes only three members, OCTN1, 2 and 3 [1,2]. However, the gene coding for OCTN3 was lost in humans [3,4], that possess only OCTN1 and OCTN2 [5]. Differently from OCTN2, which is a well-acknowledged carnitine transporter, human OCTN1 presents still uncertainties concerning the substrate specificity and, hence, the physiological role. It is expressed in several districts [6] including peritoneum, where the transporter should be involved in control of inflammatory processes that are critical in this district [7]. Transport function of OCTN1 was firstly characterized in intact cells transfected with OCTN1 cDNA [1,8,9,10], in OCTN1 cRNA injected Xenopus oocytes [9] or in membrane vesicles obtained by OCTN1 expressing cells [11]. It was defined as an organic cation transporter on the basis of specificity towards the prototype cation TEA, with a low affinity for carnitine. Then, studies performed using a metabolomic approach, highlighted transport competence for ergothioneine, a fungi metabolite [12]. Even though the high affinity towards this molecule was documented, the physiological significance of this function still remains uncertain [13,14,15]. To further investigate the function of this transporter on the single protein, the human OCTN1 was over-expressed in *E. coli* and its transport activity was assayed in proteoliposomes harboring the recombinant transporter. The specificity for TEA was confirmed and other substrates were identified such as acetylcholine and choline for which a bi-directional transport mode was demonstrated [16]. The transport sensitivity to physiological ions and effectors, suggested that the acetylcholine transport could occur in vivo mainly as efflux from cells. This also correlated to the stimulation of efflux by internal ATP and K^+^ [8,17,18]. This function was acknowledged to be possibly involved in the non-neuronal cholinergic system whose fundamental role in cells emerged in the last decades [19,20]. In this frame, the described association of the OCTN1503F variant with the Crohn’s disease could be explained by the impaired efflux of acetylcholine exhibited by this variant [16]. Studies performed in primary mesothelial [7] and HeLa cell lines [21] supported the capacity of OCTN1 in mediating acetylcholine release in vivo, as well. This finding could contribute to explain some results obtained in knockout mouse, in wild type cells or in silenced cells that could not be explained by the sole impairment of ergothioneine transport [22,23,24]. Very recently, spermine was also suggested as a physiological substrate of OCTN1 [25]. The role of OCTN1 in drug disposition and drug interaction has been well assessed [26,27,28,29,30,31,32,33,34]. Recently, it has been shown that cholesterol regulates a number of SLC transporters [35,36,37,38,39,40]. Therefore, we have investigated whether OCTN1 activity could be influenced by the presence of cholesterol in the phospholipid bilayer. To this aim, we have used two approaches, one based on cholesterol sequestration from the native membranes the other based on cholesterol addition to the artificial phospholipid bilayer of proteoliposomes.

## 2. Results

### 2.1. Effect of Cholesterol Removal on the Native or Recombinant OCTN1 

**Figure 1:**
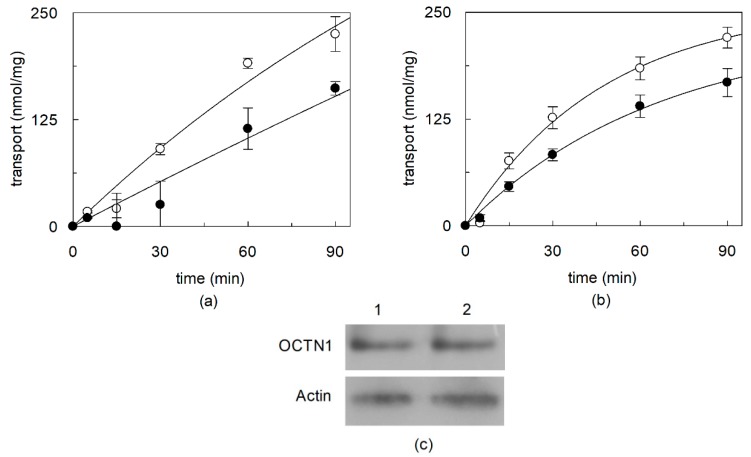
Effect of cholesterol removal on the native OCTN1. (**a**) HeLa cell protein extract or (**b**) HeLa cell sample was incubated in the absence (○) or in the presence of 10 mM MβCD (●) and then the extracts obtained in the two different conditions (**a** or **b**) and corresponding respectively to 2.1 ± 0.04 (**a**) and 2.2 ± 0.07 µg protein (**b**) were reconstituted in liposomes as described in Section 4.4. Transport was started adding 0.1 mM [^14^C]-TEA at time zero to proteoliposomes and stopped at the indicated times as described in Section 4.7. Time course data were interpolated in a first order rate equation. The values are means ± SD from three different experiments. (**c**) Immunodetected OCTN1 from HeLa cells incubated in the absence (lane 1) or in the presence (lane 2) of MβCD. Actin is shown as a control of total lysate loading. OCTN1 or Actin was immunodetected by anti-OCTN1 or anti-Actin antibody, respectively, as described in Section 4.9.

To evaluate the possible effect of cholesterol on the native human OCTN1, the protein extracted from HeLa cells was exploited. Indeed, it was previously assessed by us [21] and then also by another research group [41] that this cell line expresses a functional OCTN1 protein. OCTN1 was extracted by the non-ionic detergent Triton X-100 and reconstituted in proteoliposomes, as previously described [21]. To evaluate the possible effect of cholesterol on the transport function, the cell extract was incubated prior to reconstitution with MβCD which is widely used to sequester cholesterol from native membranes [35]. Then, MβCD-incubated or untreated HeLa extract, was reconstituted for transport assay in proteoliposomes as [^14^C]-TEA uptake. The same reconstitution procedure and transport assay was performed on cell extracts obtained from HeLa cells incubated with or without MβCD prior to solubilization (Figure 1b). To evaluate the OCTN1 specific transport activity the concentration of the protein in the cell extract was determined by a method based on western blot normalization using as a standard the recombinant OCTN1 protein [21]. Figure 1a shows that the MβCD treatment caused a decrease of the native OCTN1 activity of 35% with respect to the control, suggesting a role of cholesterol in the transport function. Similar results, with a decrease of 23% with respect to the control, were obtained by incubating cells with MβCD prior to solubilization (Figure 1b). Each time course experiment was performed with a unique proteoliposome preparation to take the protein amount and cholesterol concentration constant within a time data series.

**Figure 2:**
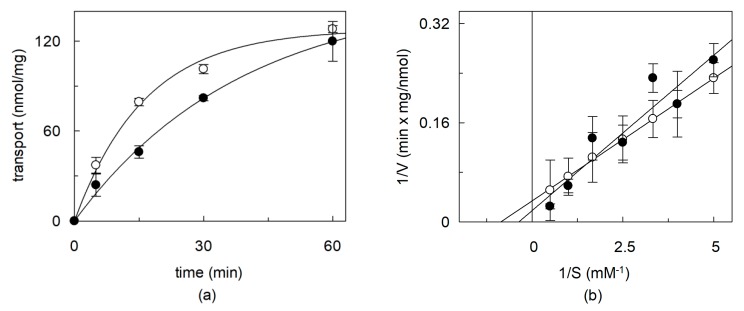
Effect of cholesterol removal from proteoliposomes on the recombinant OCTN1. The recombinant purified OCTN1 was reconstituted in liposomes as described in Section 4.5. Proteoliposomes were incubated in the absence (○) or in the presence of 10 mM MβCD (●). (**a**) Transport was started adding 0.1 mM [^14^C]-TEA at time zero to proteoliposomes and stopped at the indicated times as described in Section 4.7. Time course data were interpolated in a first order rate equation. (**b**) The transport rate was measured adding [^14^C]-TEA at the indicated concentrations to proteoliposomes. The transport reaction was stopped at 10 min, i.e., within the initial linear range of the time course. Data were plotted according to Lineweaver–Burk as reciprocal transport rate vs reciprocal TEA concentration. The values in both (**a**) and (**b**) are means ± SD from three different experiments.

To obtain more reliable information on the effect of cholesterol on the hOCTN1, the recombinant protein was exploited, whose transport features overlap those of the native protein [21]. Before testing the effect of addition of cholesterol to the cholesterol-free recombinant protein, the cholesterol concentration in the phospholipid preparations used for reconstitution was measured by an enzymatic assay (see Material and methods). 10 µg/ mg total lipids corresponding to 1.0% cholesterol in the phospholipid mixture [7,21] used for reconstitution was calculated. The effect of incubation of the lipids with MβCD was firstly assessed, by adding MβCD to the proteoliposome preparation. After this treatment, the concentration of cholesterol was lowered to 6.5 µg/mg total lipids corresponding to 0.65% cholesterol. After removal of MβCD, transport was measured as TEA uptake. As shown by Figure 2a the MβCD treatment caused a decrease of transport, with respect to the control. This correlated well with the presence of cholesterol in the phospholipid mixture and with the data of Figure 1 obtained with the native protein. To gain information on the effect of cholesterol removal on the properties of the transporter, the kinetics of transport catalyzed by the recombinant protein were analyzed studying the dependence of the transport rate on the substrate concentration. Data plotted according to Lineweaver–Burk (Figure 2b) showed a transport impairment at lower TEA concentration, while at higher concentration the transport rate increased. This anomalous behavior resulted in an increase of both the Vmax and the Km. The catalytic efficiency (Vmax/Km) decreased from 25.3 to 19.8 (ml ⋅ min^−1^ ⋅ mg^−1^) after the treatment with MβCD.

### 2.2. Dependence of CHS Addition on the Recombinant OCTN1

**Figure 3:**
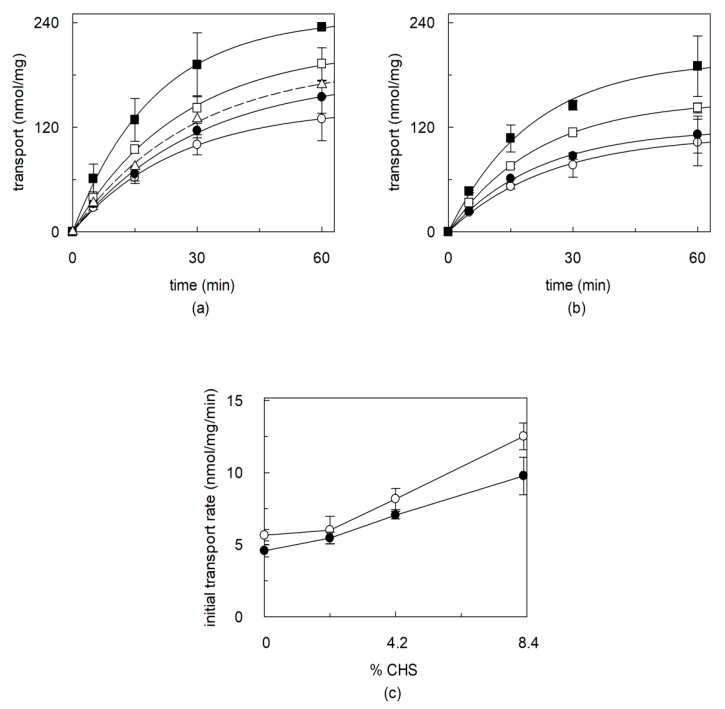
Effect of increasing cholesterol (CHS) concentrations on OCTN1 mediated transport of TEA and acetylcholine. The recombinant OCTN1 was reconstituted in liposomes as described in Section 4.5. except that (**a** and **b**) proteoliposomes batches used for each time course experiment were reconstituted in the presence of 0 (○), 2.1 (●), 4.2 (□), 8.3 (■) % CHS (corresponding to 0, 21, 42 and 83 µg CHS/mg total lipids, respectively). (**a**) Transport was started adding 0.1 mM [^14^C]-TEA (**b**) or 0.1 mM [^3^H]-acetylcholine, at time zero to proteoliposomes and stopped at the indicated times as described in Section 4.7. Time course data were interpolated in a first order rate equation. The values are means ± SD from three different experiments. (**c**) Transport rates of [^14^C]-TEA (○) or [^3^H]-acetylcholine (●) uptake, calculated as K x transport at equilibrium from (**a**,**b**), reported as function of the % CHS.

The dependence of the transport activity on cholesterol concentration was then studied on the recombinant OCTN1. To this aim, CHS, a widely used cholesterol analogue which is more suitable than free cholesterol for incorporating into membranes [35], was added to the phospholipid mixture. In these experiments, besides TEA, transport of acetylcholine was also measured, that is a physiological substrate of OCTN1 [5,15,20,42]. Figure 3a,b show the time courses of TEA and acetylcholine uptake, respectively, in the presence of increasing CHS concentrations. As in Figure 1, each time course experiment was performed with a unique proteoliposome preparation to avoid variations of both protein and CHS amount within a data time series. The transport of both substrates increased up to about double the control (without added cholesterol) at a CHS concentration of 8.3% (83 µg CHS/ mg total lipids). This value does not include the 1% cholesterol initially present in the phospholipid preparations. Initial transport rates for TEA and acetylcholine, calculated as the product of k (the first order rate constant) and the transport at equilibrium from Figure 3a,b, are reported in Figure 3c. The rate of transport nearly doubled at the highest cholesterol concentration with respect to the control for either the substrates. Inclusion of more cholesterol into proteoliposomes caused a decrease of transport (Figure 3a, dotted line). 

**Figure 4:**
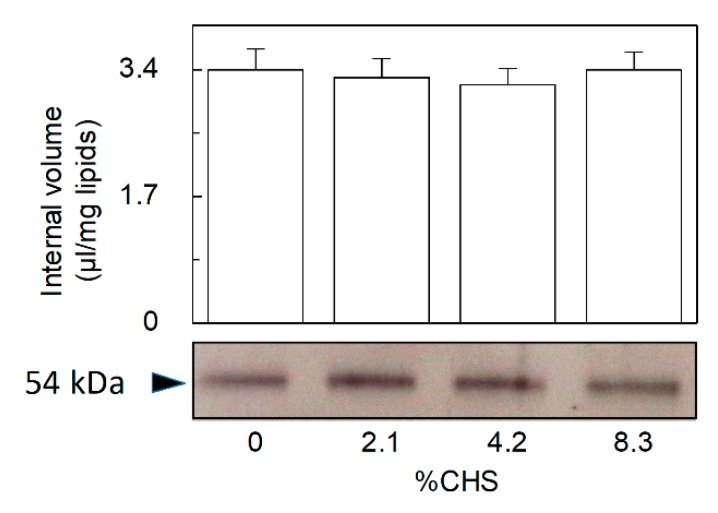
Effect of CHS on the internal volume of the proteoliposomes. The recombinant OCTN1 was reconstituted in liposomes as described in Section 4.5. except that in proteoliposomes batches used for each time course experiment were reconstituted in the presence of 0 (○), 2.1 (●), 4.2 (□), 8.3 (■) % CHS (corresponding to 0, 21, 42, and 83 µg CHS/mg total lipids, respectively). Internal volume of proteoliposomes was measured as described in Section 4.5. (upper panel). Immunodetected OCTN1 bands (anti-His antibody, as described in Section 4.9) from the different proteoliposomes preparations (lower panel). The relative concentration of the immuno-stained proteins, normalized to the 0 % CHS (considered as 100%) was 109 ± 14% (2.1% CHS); 104 ± 5.8% (4.2% CHS); 106 ± 9.1% (8.3% CHS), as derived from three different experiments (± S.D.).

The initial transport rate gives information on the specific activity of the transporter, while the transport at equilibrium can be correlated to the number or to the size of proteoliposomes, i.e., the fraction of vesicles harboring the active transporter incorporated into the membrane with respect to the total liposome vesicles. To discriminate between the two possibilities, the intraliposomal volume and the protein incorporated into the proteoliposomes were measured. The internal volumes ranged from 3.2 and 3.4 µL/mg phospholipids in the various samples with no significant variations (Figure 4, upper panel). Therefore, CHS did not cause variations in the proteoliposome volume. Surprisingly no changes in incorporated purified OCTN1 protein detected by a specific antibody were found, too (Figure 4, lower panel). 

Thus, the increase in transport at equilibrium shown in Figure 3a,b could only be ascribed to an increased fraction of active (appropriately folded) protein induced by the presence of CHS. This also correlates with the increased transport rate associated with the addition of CHS to proteoliposomes. 

**Figure 5:**
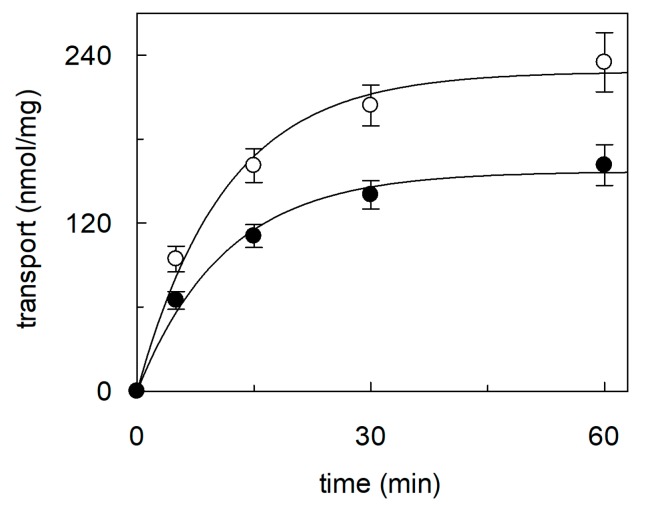
Effect of removal of CHS after addition to proteoliposomes. The recombinant OCTN1 was reconstituted in liposomes as described in Section 4.5. except that in the presence of 8.3% CHS corresponding to 83 µg CHS/mg total lipids. Proteoliposomes were incubated in the absence (○) or in the presence of 10 mM MβCD (●). Transport was started adding 0.1 mM [^14^C]-TEA at time zero to proteoliposomes and stopped at the indicated times as described in Section 4.7. Time course data were interpolated in a first order rate equation. The values are means ± SD from three different experiments.

As a further proof of the relationships between cholesterol concentrations and protein activity, proteoliposomes prepared with the optimal CHS concentration were then incubated with MβCD to remove the CHS previously incorporated. A decrease in OCTN1 activity was detected in proteoliposomes after MβCD treatment. Also, in this case, the effect was observed both on the initial transport rate and on the transport at equilibrium (Figure 5). 

To confirm the CHS removal after MβCD treatment, CHS concentration was measured (see the method in Section 4.6) and compared to that of the untreated sample. The initial concentration of CHS of 8.3% (83 µg CHS/mg total lipids) was reduced to 5% (50 µg CHS/mg total lipids) corresponding to a reduction of 40% of the initial CHS concentration. The decrease of CHS by MβCD was comparable to the 31% decrease in transport activity observed in Figure 5.

### 2.3. Effects of CHS on Regulation of OCTN1 by Physiological or Non-Physiological Effectors

**Figure 6:**
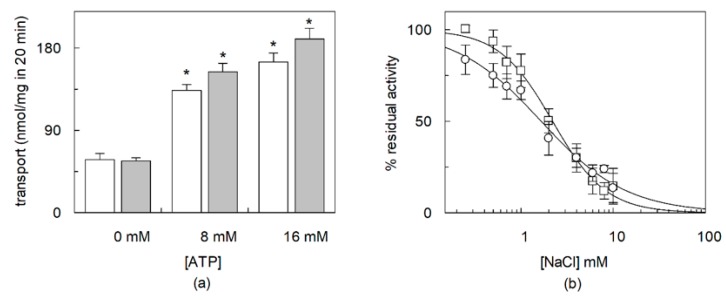
Effect of CHS on regulation of OCTN1 by ATP or NaCl. The recombinant OCTN1 was reconstituted in liposomes as described in Section 4.5. except that in the presence of 8.3% CHS corresponding to 83 µg CHS/mg total lipids and (**a**) in the presence of the indicated ATP concentrations. Transport was started adding 0.1 mM [^14^C]-TEA (white column, ○) or [^3^H]-acetylcholine (grey column, □) at time zero to proteoliposomes. (**b**) Increasing NaCl extraliposomal concentration were added together to the radioactive substrates. The transport reaction was stopped at 20 min. Percent residual activity with respect to the control data were interpolated in an IC_50_ equation (Dose-response curves). The values are means ± SD from three experiments. Significantly different for * *p* < 0.01 as calculated from Student’s t test analysis.

It was previously reported that OCTN1 is regulated by intraliposomal (intracellular) ATP [8,17]. Thus, the effect of ATP was tested on the protein treated with the optimal CHS concentration. As shown by Figure 6a, ATP stimulated the transport when measured either as TEA or acetylcholine uptake. The possible influence of cholesterol was also tested on the hOCTN1 regulation by external Na^+^ which inhibits the transport. The dose-response of the external Na^+^ effect on [^14^C]-TEA or [^3^H]-acetylcholine uptake was studied (Figure 6b) and an IC_50_ value of 1.7 ± 0.5 or 2.2 ± 0.2 mM, respectively for TEA or acetylcholine was derived. Thus, the OCTN1 added with CHS is regulated by ATP and Na^+^ in a similar fashion as the transporter reconstituted under basic conditions (no CHS added) [16,17].

Inhibition of OCTN1 by HgCl_2_ due to interaction of the metal with Cys residues was previously described [43]. The addition of CHS did not change the mercurial inhibitory effect. The measured IC_50_ value was 0.27 ± 0.05 mM or 0.2 ± 0.03 mM, respectively in the absence or in the presence of CHS (Supplementary Figure S1), indicating that cholesterol does not interfere with the accessibility of the Cys residues to the Hg^++^.

**Figure 7:**
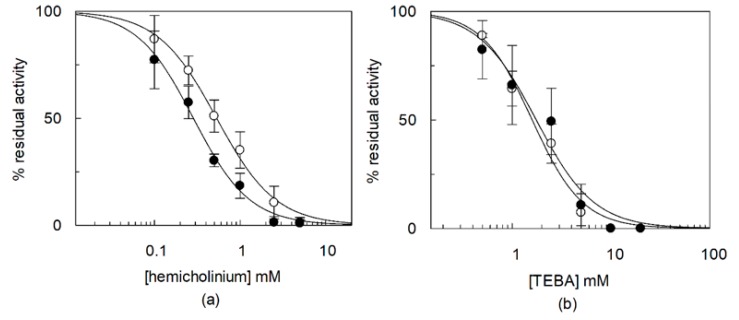
Effect of hemicholinium and TEBA on the OCTN1 activity measured in the presence or absence of CHS. The recombinant OCTN1 was reconstituted in liposomes as described in Section 4.5. except that in the absence (○) or in the presence (●) of 8.3% CHS corresponding to 83 µg CHS/mg total lipids. (**a**) Transport was started adding 0.1 mM [^3^H]-acetylcholine at time zero to proteoliposomes in the presence of increasing external hemicholinium concentrations (0-0.1-0.3-0.5-0.8-1-1.5-2.6 mM) or (**b**) adding 0.1 mM [^14^C]-TEA in the presence of increasing external TEBA concentrations (0-0.5-1-2.5-5-10-20 mM). The transport reaction was stopped at 20 min. Percent residual activity with respect to the control data were interpolated in an IC_50_ equation (Dose-response curves). The values are means ± SD from three experiments.

The effect of the acetylcholine analogue hemicholinium or the TEA analogue TEBA was studied on the recombinant hOCTN1 in the absence or in the presence of CHS. To this aim, [^3^H]-acetylcholine or [^14^C]-TEA uptake, respectively, was measured in the presence of increasing concentrations of the analogues (Figure 7a,b). The dose-response analysis revealed an IC_50_ for hemicholinium of 0.53 ± 0.046 mM or 0.30 ± 0.052 mM, and an IC_50_ for TEBA of 1.6 ± 0.3 mM or 1.9 ± 0.7 mM, respectively in the absence or in the presence of CHS.

### 2.4. Effects of CHS on the Kinetics of OCTN1 Mediated Transport

**Figure 8:**
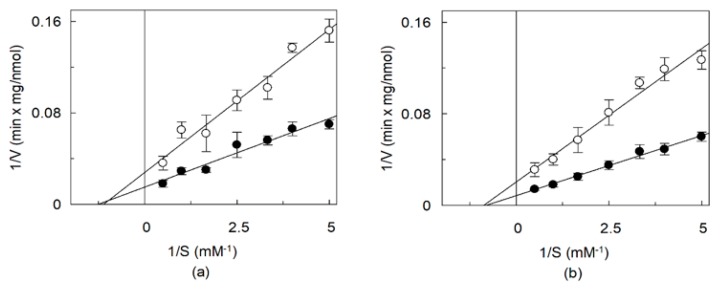
Effect of CHS on OCTN1 transport kinetics. The recombinant OCTN1 was reconstituted in liposomes as described in Section 4.5. except that in the absence (○) or in the presence (●) of 8.3% CHS corresponding to 83 µg CHS/mg total lipids. The transport rate was measured adding [^14^C]-TEA (**a**) or [^3^H]-acetylcholine (**b**) at the indicated increasing concentrations to proteoliposomes. The transport reaction was stopped at 10 min. Data were plotted according to Lineweaver–Burk as reciprocal transport rate vs reciprocal TEA (**a**) or acetylcholine (**b**) concentration. The values are means ± SD from three experiments.

Kinetic analysis was performed varying TEA or acetylcholine concentration (Figure 8a,b), in the absence or in the presence of CHS. Double reciprocal (Lineweaver–Burk) plot analysis showed that the presence of CHS doubled the Vmax of transport both in the case of TEA and acetylcholine. The Vmax of TEA transport increased from 36.1 ± 8.6, in absence of CHS, to or 66.6 ± 16.4 nmol ⋅ min^−1^ ⋅ mg protein^−1^, in the presence of CHS. The Vmax of acetylcholine transport increased from 49.3 ± 12.4, in absence of CHS to 119 ± 19.8 nmol ⋅ min^−1^ ⋅ mg protein^−1^, in the presence of CHS. While, no significant differences of Km were found. The Km for TEA was 0.9 ± 0.29 or 0.8 ± 0.28 mM in the absence or presence of CHS, respectively. The Km for acetylcholine was 1.1 ± 0.37 or 1.25 ± 0.27 mM in the absence or in the presence of CHS, respectively [35].

### 2.5. In Silico Analysis of the Interaction of OCTN1 with Cholesterol 

**Figure 9:**
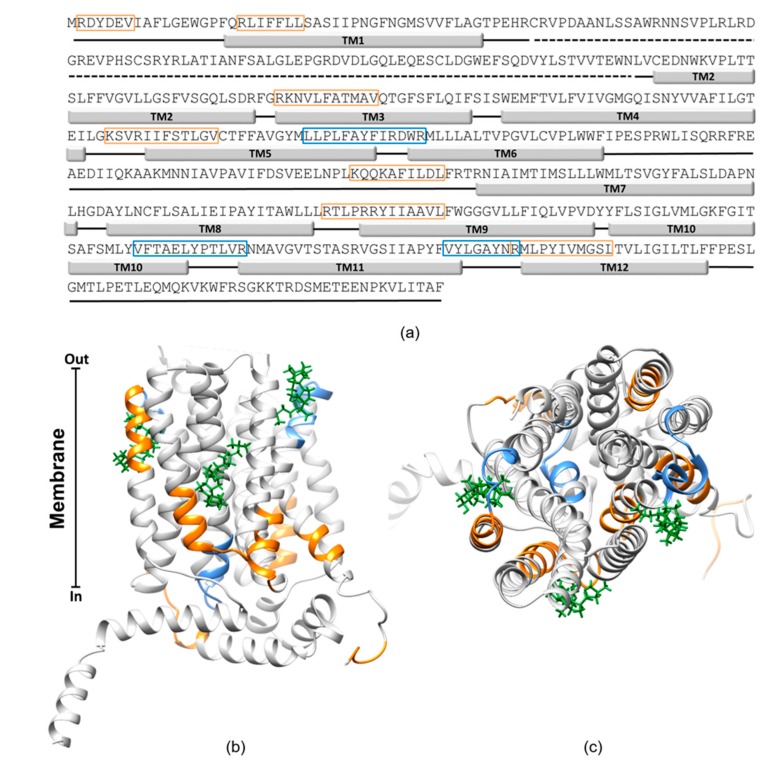
Bioinformatic analysis. (**a**) Primary structure of the human OCTN1 transporter. The transmembrane α-helices (TM1-12) are depicted as light gray rectangles. The region containing the amino acids from 50 to 133 (big extracellular loop, absent in the homology model) is depicted as dotted line. Hypothetical cholesterol-binding motifs CRAC and CARC are indicated as blue and orange boxes, respectively. (**b**,**c**) side and top view, respectively, of the homology structural model of the OCTN1 docked with three molecules of cholesterol (green). In this ribbon representation, only the best three (lowest binding energy) out of the twenty-two predicted by Achilles blind docking server, are reported.

To verify if cholesterol could interact with OCTN1, CARC and CRAC motifs were searched in the sequence. These sequences are acknowledged as cholesterol-binding motifs in membrane proteins [44]. As shown in Figure 9a, several of these motifs are present in the OCTN1 sequence and most of these motifs overlap to the predicted transmembrane segments, highlighted by solid rectangles. A prediction of the cholesterol interaction sites based on docking analysis was performed using the OCTN1 homology model previously obtained [5]. Many cholesterol poses were predicted by the docking, with a wide range of interaction energies. However, it is well known that in the presence of strong hydrophobic interactions, as in the case of cholesterol, docking procedures are not always straightforward [45]. Therefore, only those poses which fulfill both higher scores (lower binding energy) and the vicinity to some CARC-CRAC motifs were shown in the figures (Figure 9b,c). The cholesterol molecules are located at the interface between some hydrophobic moieties of the protein and the membrane. As expected, this lipid may mediate the interactions between the protein and the membrane in agreement with the stimulation of the transport function. 

## 3. Discussion

To describe the role of cholesterol on the function of hOCTN1 we used the proteoliposome experimental tool that is the most suitable for easily changing the lipid composition of the membrane. However, cholesterol is difficult to insert into the phospholipid bilayer of proteoliposomes. Thus, the widely used soluble analogue CHS [35] has been included in the proteoliposome preparations harboring the cholesterol-free recombinant hOCTN1 expressed in *E. coli*. Together with this strategy, the native transporter extracted from HeLa cells was exploited. Starting from the hypothesis that the protein extracted from cells harbors a native cholesterol shell, the cholesterol sequestering agent MβCD was used to remove, at least partially, the native membrane cholesterol as previously described [35,36,38,40]. Interestingly, the removal/reduction of the cholesterol shell from the native protein or the addition of cholesterol (CHS) to the cholesterol-free protein gave opposite effects, i.e., impairment or stimulation of transport, respectively. Even though we cannot exclude the presence of some differences between CHS and cholesterol effects, these findings concur in demonstrating that cholesterol is required for a proper functioning of the OCTN1 transporter and that the lipid has a physiological role, as found for other transporters [35,36,38,40,46,47,48]. Interestingly, it came evident that a small fraction of cholesterol, measured by an enzymatic assay, is present in the lipid mixture used for proteoliposome assembly. This explains why a functionally active OCTN1 was found in proteoliposomes reconstituted with the recombinant (cholesterol-free) protein without adding CHS, as well (Figure 2). This finding correlates with previous works describing the OCTN1 function in proteoliposomes [16,17]. However, the functional protein in absence of CHS accounts for less than 50% of the full activity, even though the Km and the main regulatory properties such as the ATP stimulation or the Na^+^ inhibition are virtually identical. Addition of further cholesterol, as CHS, increases the transport activity of OCTN1, about doubling the amount of functional protein under optimal condition of CHS/phospholipid mixture. Altogether the collected experimental results indicate that the protein is in its functional state in the presence of cholesterol. Thus, addition of CHS increases the number of active proteins. This correlates well with the kinetic analysis that shows an increase of the Vmax of transport while the Km is not influenced. It might be that cholesterol participates to the folding of the protein also mediating the correct positioning of some hydrophobic transmembrane segments of OCTN1 in the membrane, according to the acknowledged role of this lipid on membrane proteins [44]. Indeed, the computational analysis predicts the cholesterol poses in some locations that are at the boundary between the hydrophobic stretches of the protein and the membrane (Figure 9b). The effect of cholesterol on OCTN1 correlates with previous findings on SLC38A9, SLC7A5 (LAT1), SLC1A5 (ASCT2) and SLC22A2 (OCT2) transporters [35,36,38,40]. Similarities as well as differences emerge from this work in the mechanism of action of cholesterol with respect to the other transporters. The increase of Vmax seems to be a common finding for the transporters characterized so far [35,38,39,40,49,50]. While, mostly invariant Km was found for OCTN1 as well as, ASCT2 and SLC38A9. Clearly different effects of cholesterol on the substrate affinity were observed in the case of OCT2 [40]. Indeed, OCT2 showed a strong variation of substrate affinity and changes in the allosteric binding of 1-methyl-4-phenylpyridinium (MPP^+^). While, OCTN1 shows no or very small changes in Km as well as in regulation by several effectors, including TEBA and hemicholinium that have been tested on OCTN1 for the first time in this work. The differences between OCTN1 and OCT2 may be unexpected since the two proteins belong to the same SLC22 family. However, such differences correlate with the relatively low identity of 32.3% existing between the sequences of the two proteins, (see Supplementary Figure S2). This also correlates with different locations of some CRAC-CARC motifs (see Supplementary Figure S2 in comparison with Figure 9a), which should be the sites of interaction of cholesterol. Interestingly, a more extensive removal of cholesterol from proteoliposomes harboring the recombinant OCTN1, leads to a small variation of the Km (Figure 2b) that can be observed also in the case of LAT1, even though in an opposite fashion: a Km increase (reduced affinity) for OCTN1, a Km decrease (increased affinity) for LAT1. However, in both cases the variations have a scarce significance, thus, this finding indicates a limited action of cholesterol on the conformation of the substrate binding sites of these transporters, differently from OCT2. Thus, except than for OCT2, it appears that cholesterol essentially increases the rate of the conformational changes underlying the transport path, in line with an action on improving the folding of the proteins. This might be in agreement with the predicted interaction of more than one cholesterol molecule with each protein and with the location of cholesterol molecules at the interface between transmembrane α-helices and the membrane, as suggested by the computational analysis for both LAT1 and OCTN1. The described results open new perspectives in the study of the role of cholesterol in cancer cells in which the OCTN1 transporter is expressed and may play important roles in inflammatory and/or immunomodulatory processes.

## 4. Materials and Methods

### 4.1. Materials 

Amberlite XAD-4, egg yolk phospholipids (3-sn-phosphatidylcoline 60%), CHS, Triton-X100, Sephadex G-75, TEA and acetylcholine were purchased from Merck Life Science s.r.l., 20149 Milano, Italy; tetraethylammonium bromide [ethyl-1-^14^C] was from ARC (American Radiolabeled Chemicals, St. Louis, MO 63146 USA); acetylcholine iodide [acetyl-^3^H] was from Perkin-Elmer Italia S.p.A. 20126 Milano, MI, Italy. All the other reagents were of analytical grade.

### 4.2. CHS Solubilization 

CHS was solubilized in 20 mM Tris/HCl (pH 8.8) and 5% Triton X-100 by two sonication cycles (2 min, no pulse, 40 W) with a Vibracell VCX-130 sonifier (Sonics, 53 Church Hill Road, Newtown, CT 06470, USA). Solution was centrifuged for 5 min at 10.000 g. The supernatant was then used for the reconstitution procedure. The pH was adjusted to pH 7.5 before reconstitution.

### 4.3. Cell culture 

HeLa cells were maintained in Dulbecco’s Modified Eagle Medium (DMEM) supplemented with 10% (*v*/*v*) fetal bovine serum (FBS, GIBCO, ThermoFisher Scientific, Milan, Italy), 1 mM glutamine, 1 mM sodium pyruvate and Pen/strep as antibiotics. Cells were grown on 10 cm^2^ plates at 37 °C in a humidified incubator and a 5% CO_2_ atmosphere.

### 4.4. Reconstitution of the OCTN1 Transporter from HeLa Cell Extract into Proteoliposomes

HeLa cells pellet was treated with 150 µL TX-100 10%. After vortexing cells have been incubated 30 min on ice and then centrifuged 10 min 12.000× *g* at 4 °C. The cell membrane extract (supernatant) was used for the reconstitution [21]. 30 µL supernatant were included to the reconstitution mixture containing 120 µL 10% egg yolk phospholipids in the form of sonicated liposomes prepared as previously described [51], 80 µL 10% Triton X-100 solution, 16 mM ATP, 7 µL of 500 mM Tris/HCl (pH 7.5) in a final volume of 700 μL.

### 4.5. Reconstitution of the Recombinant OCTN1 Transporter into Liposomes

OCTN1 over-expressed in *E. coli* and purified as previously described [17] was used for reconstitution in liposomes. The composition of the initial mixture used for reconstitution (except when differently indicated) was 200 µL of the purified protein (5 µg protein in 0.05% DDM), CHS at the indicated concentrations solubilized in 240 µL of 20 mM Tris/HCl (pH 7.5) and 5% Triton X-100 or buffer and Triton X-100 alone in the case of samples without CHS, 120 µL of 10% egg yolk phospholipids in the form of sonicated liposomes prepared as previously described [51], 16 mM ATP and (where indicated) in a final volume of 700 μL. The purified OCTN1 inserted in liposomal membrane by removing the detergent from mixed micelles containing detergent, protein, and phospholipids with Amberlite XAD-4 in a batch-wise procedure. Where indicated, proteoliposome internal volume was measured using a colorimetric phosphate method [52]. In detail, 30 mM dipotassium phosphate (K_2_HPO_4_) was added to the reconstitution mixture. After elution from Sephadex G75 pre-equilibrated with 5 mM Tris/HCl (pH 7.5), 100 µL of sample were used for the colorimetric reaction. A solution containing 150 µL of 10% SDS was used to solubilize liposomal membranes. Then, 700 µL of a solution prepared with 10 mM hexammonium heptamolybdate 4-hydrate, 0.3 mM H_2_SO_4_ and 0.1 mM FeSO_4_ was added starting an incubation of 30 min in the dark. At the end, absorbance from each sample was measured at 578 nm. Internal volume in µL was derived from nmol of phosphate incorporated in liposomes.

### 4.6. MβCD Treatment

Each HeLa cell sample or HeLa cell extract was incubated with 10 mM MβCD for 90 min at 37 °C under stirring. Then, the respective extracts were reconstituted in proteoliposomes.

Proteoliposomes reconstituted with the recombinant OCTN1 and added or not with CHS were incubated with MβCD as above described and then, MβCD was removed by chromatography on Sephadex G-75 column as described in Section 4.7.

### 4.7. Transport Measurements

Proteoliposomes (550 µL) were passed through a Sephadex G-75 column (0.7 cm diameter x 15 cm height) pre-equilibrated with 5 mM Tris/HCl (pH 7.5). The first 550 µL turbid eluate was collected and divided in aliquots (samples) of 100 µL each. Transport was started by adding 0.1 mM [^14^C]-TEA or [^3^H]-acetylcholine to the proteoliposome samples and stopped by adding 0.5 mM HgCl_2_ at the desired time interval. In control samples, the inhibitor was added at time zero according to the inhibitor stop method [53]. Finally, each sample of proteoliposomes (100 µL) was passed through a Sephadex G-75 column (0.6 cm diameter × 8 cm height) in order to separate the external from the internal radioactivity. Liposomes were eluted with 1 mL of 50 mM NaCl and collected in 4ml of scintillation mixture, vortexed, and counted. The experimental values were corrected by subtracting the respective controls [16,17]. The uptake time course data were interpolated using a first order rate equation from which rate constants were derived as the product of k (the first order rate constant) and the transport at the equilibrium. Data from dose-response experiments were fitted into an IC_50_ equation. For kinetic experiments the initial rate of transport expressed as nmol ⋅ min^−1^ ⋅ mg protein^−1^, was measured by stopping the reaction after 10 min, i.e., within the initial linear range of [^14^C]-TEA or [^3^H]-acetylcholine uptake into the proteoliposomes. Kinetic constants were estimated from interpolation of the experimental data into a Lineweaver–Burk equation. The Grafit (version 5.0.3) software was used for calculations.

### 4.8. Quantitation of Cholesterol

Quantitation was performed using the Cholesterol Quantitation Kit MAK043 by Merck Life Science s.r.l., 20149 Milan, Italy,). Assay was performed in the presence of cholesterol esterase with absorbance measured on a microplate reader at 570 nm.

### 4.9. Electrophoretic and Western Blotting Analysis

Protein amount was measured by densitometry of SDS-PAGE stained by Coomassie Blue, using the ChemiDoc imaging system equipped with Quantity One software (Bio-Rad Laboratories) [54]. Immunoblotting analysis was performed using anti-His antibody 1:1000 Merck Life Science s.r.l., 20149 Milan, Italy,), Rabbit anti-OCTN1 antibody [21] or Mouse Monoclonal β actin Antibody 1: 2000 (Sigma) incubated 1h in 3% BSA under shaking at room temperature and then revealed by chemiluminescence assay (Amersham^TM^ ECL^TM^ Prime Western Blotting Detection Reagent, GE Healthcare s.r.l., Milan, Italy) in the case of anti-His or after incubation with the secondary antibody, anti-rabbit or anti-mouse IgG, 1:2000 respectively (Cell Signalling, EuroClone, Pero (Milan), Italy). 

### 4.10. Docking

The homology structural model of the hOCTN1 was built by Iterative Threading ASSEmbly Refinement, (I-TASSER) available at https://zhanglab.ccmb.med.umich.edu/I-TASSER/. The large hydrophilic loop between the transmembrane domain I and II (amino acids 42-141) was removed from the structure by Chimera 1.13.1 software [41]. The 3D structure of cholesterol was downloaded from PubChem Database (https://pubchem.ncbi.nlm.nih.gov/#) as .sdf file and saved as .pdb by Chimera. The ligand and the transporter protein were prepared using ADT [55]. Gasteiger charge was assigned and the files were saved in PDBQT format. Achilles blind docking server available at https://bio-hpc.ucam.edu/achilles/entry was used to predict possible cholesterol-binding sites. AutoDock Vina was used [56].

## Supplementary Materials

Supplementary materials can be found at https://www.mdpi.com/1422-0067/21/3/1091/s1.

## Abbreviations

OCTN	Novel Organic Cation Transporters
SLC	SoLute Carrier
CHS	Cholesteryl HemiSuccinate
MβCD	Methyl-beta-CycloDextrin
DDM	n-Dodecyl-β-D-maltoside
TEA	Tetraethylammonium
TEBA	Benzyltriethylammonium
