# Supplementary material for: Effect of Cholesterol on the Organic Cation Transporter OCTN1 (SLC22A4)
Source: Int J Mol Sci. 2020 Feb 6;21(3):1091. doi: 10.3390/ijms21031091 (PMC7037232; doi:10.3390/ijms21031091)
Supplement: Supplementary file 1 [file ijms-21-01091-s001.pdf]

International Journal of *Molecular Sciences*

supplementary

Effect of Cholesterol on the Organic Cation Transporter OCTN1 (SLC22A4)

Lorena Pochini, Gilda Pappacoda, Michele Galluccio, Francesco Pastore, Mariafrancesca Scalise, and Cesare Indiveri*

Department of Biology, Ecology and Earth Sciences (DiBEST), Unit of Biochemistry and Molecular Biotechnology, University of Calabria, via P. Bucci 4c, 87036 Arcavacata di Rende, Italy; lorena.pochini@unical.it (L.P.); gilda.pappacoda@gmail.com (G.P.); michele.galluccio@unical.it (M.G.); francesco.pastore92@gmail.com (F.P.); mariafrancesca.scalise@unical.it (M.S.); cesare.indiveri@unical.it (C.I.).

* Correspondence: cesare.indiveri@unical.it; Tel.:+39-0984-492939

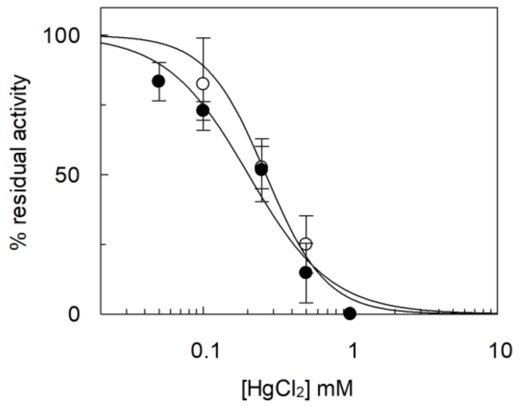

Supplementary Figure 1. Effect of CHS on the inhibitory effect by $HgCl_2$ on the hOCTN1. The recombinant hOCTN1 was reconstituted in liposomes as described in section 4.5. except that in the absence (○) or in the presence (●) of 8.3 % CHS corresponding to 83 μg CHS/mg total lipids. Transport was started adding 0.1 mM [^{14}C]-TEA at time zero to proteoliposomes in the presence of increasing $HgCl_2$ concentrations (0-0.05-0.1-0.25-0.5-1 mM). The transport reaction was stopped at 20 minutes. Percent residual activity with respect to the control data were interpolated by an IC_{50} equation (Dose-response curves). The values are means ± SD from three experiments.

```
OCTN1   -MRDYDEVIAFLGEWGPFQRLIFFLLSASIIPNGFNGMSVVFLAGTPEHRCRVPDAANLSS--AWR-----NNSVPLRLR
OCT2    MPTTVDDVLEHGGEFHFFQKQMFFLLALLSATFAPIYVGIVFLGFTPDHRCRSPGVAELSLRCGWSPAEELNYTVPGPGP

OCTN1   DGREVPHSCSRYRLAT-IANFSALGLEPGRDVDLGQLEQESCLDGWEFSQDVYLSTVVTEWNLVCEDNWKVPLTTSLFFV
OCT2    AGEASPRQCRRYEVDWNQSTFDCVDPLASLDTNRSRLPLGPCRDGWVYETPG--SSIVTEFNLVCANSWMLDLFQSSVNV

OCTN1   GVLLGSFVSGQLSDRFGRKNVLFATMAVQTGFSFLQIFSISWEMFTVLFVIVGMGQISNYVVAFILGTEILGKSVRIIFS
OCT2    GFFIGSMSIGYIADRFGRKLCLLTTVLINAAAGVLMAISPTYTWMLIFRLIQGLVSKAGWLIGYILITEFVGRRYRRTVG

OCTN1   TLGVCTFFAVGYMLLPLFAYFIRDWRMLLLALTVPGVLCVPLWWFIPESPRWLISQRRFREAEDIIQKAAKMNNIAVPAV
OCT2    -IFYQVAYTVGLLVLAGVAYALPHWRWLQFTVSLPNFFFLLYYWCIPESPRWLISQNKNAEAMRIIKHIAKKNGKSLPAS

OCTN1   IFDSVEELNPLKQQKAFILDLFRTRNIAIMTIMSLLLWMLTSVGYFALSLDAPNLHGDAYLNCFLSALIEIPAYITAWLL
OCT2    LQRLRLEEETGKKLNPSFLDLVRTPQIRKHTMILMYNWFTSSVLYQGLIMHMGLAGDNIYLDFFYSALVEFPAAFMIILT

OCTN1   LRTLPRRYIIAAVLFWGGGVLLFIQLVPVDYYFLSIGLVMLGKFGITSAFSMLYVFTAELYPTLVRNMAVGVTSTASRVG
OCT2    IDRIGRRYPWAASNMVAGAACLASVFIPGDLQWLKIIISCLGRMGITMAYEIVCLVNAELYPTFIRNLGVHICSSMCDIG

OCTN1   SIIAPYFVY-LGAYNRMLPYIVMGSLTVLIGILTLFFPESLGMTLPETLEQMQKVKWFRSGKKTRDSMETEENPKVLITA
OCT2    GIITPFLVYRLTNIWLELPLMVFGVLGLVAGGLVLLLPETKGKALPETIEEAENMQRPRKNKEKMIYLQVQKLDIPLN--

OCTN1   F
OCT2    -
```

Supplementary Figure 2. CRAC and CARC cholesterol binding motif conservation. The human OCTN1 and OCT2 protein sequences were aligned by ClustalX software. CRAC and CARC motif, conserved across the two proteins, are indicated as shadowed blue and orange boxes, respectively.
